# Persistent PTSD symptoms are associated with plasma metabolic alterations relevant to long-term health: A metabolome-wide investigation in women

**DOI:** 10.1017/S0033291724003374

**Published:** 2025-02-10

**Authors:** Yiwen Zhu, Katherine H. Shutta, Tianyi Huang, Raji Balasubramanian, Oana A. Zeleznik, Clary B. Clish, Julián Ávila-Pacheco, Susan E. Hankinson, Laura D. Kubzansky

**Affiliations:** 1 Department of Epidemiology, Harvard T.H. Chan School of Public Health, Boston, MA, USA; 2 Department of Biostatistics, Harvard T.H. Chan School of Public Health, Boston, MA, USA; 3 Channing Division of Network Medicine, Department of Medicine, Brigham and Women’s Hospital and Harvard Medical School, Boston, MA, USA; 4 Harvard Medical School, Boston, MA, USA; 5 Department of Biostatistics and Epidemiology, School of Public Health and Health Sciences, University of Massachusetts Amherst, Amherst, MA, USA; 6 Broad Institute of Massachusetts Institute of Technology and Harvard, Cambridge, MA, USA; 7 Department of Social and Behavioral Sciences, Harvard T.H. Chan School of Public Health, Boston, MA, USA

**Keywords:** metabolomics, PTSD, distress, Nurses’ Health Study II, metabolic alterations, women’s health

## Abstract

**Background:**

Post-traumatic stress disorder (PTSD) is characterized by severe distress and associated with cardiometabolic diseases. Studies in military and clinical populations suggest that dysregulated metabolomic processes may be a key mechanism. Prior work identified and validated a metabolite-based distress score (MDS) linked with depression and anxiety and subsequent cardiometabolic diseases. Here, we assessed whether PTSD shares metabolic alterations with depression and anxiety and if additional metabolites are related to PTSD.

**Methods:**

We leveraged plasma metabolomics data from three subsamples nested within the Nurses’ Health Study II, including 2835 women with 2950 blood samples collected across three time points (1996–2014) and 339 known metabolites assayed by mass spectrometry-based techniques. Trauma and PTSD exposures were assessed in 2008 and characterized as follows: lifetime trauma without PTSD, lifetime PTSD in remission, and persistent PTSD symptoms. Associations between the exposures and the MDS or individual metabolites were estimated within each subsample adjusting for potential confounders and combined in random-effects meta-analyses.

**Results:**

Persistent PTSD symptoms were associated with higher levels of the previously developed MDS. Out of 339 metabolites, we identified 29 metabolites (primarily elevated glycerophospholipids and glycerolipids) associated with persistent symptoms (false discovery rate < 0.05; adjusting for technical covariates). No metabolite associations were found with the other PTSD-related exposures.

**Conclusions:**

As the first large-scale, population-based metabolomics analysis of PTSD, our study highlighted shared and distinct metabolic differences linked to PTSD versus depression or anxiety. We identified novel metabolite markers associated with PTSD symptom persistence, suggesting further connections with metabolic dysregulation that may have downstream consequences for health.

## Introduction

Post-traumatic stress disorder (PTSD) is a debilitating psychiatric disorder of severe distress and occurs after experiencing or witnessing a traumatic event. The lifetime prevalence of PTSD is ~7–8% among US adults, and women are more than twice as likely to develop PTSD compared to men (Kilpatrick et al., 2013). PTSD is associated with adverse physical health, including elevated risks of cardiometabolic conditions (Sumner et al., 2015), certain cancers, and early mortality (Roberts et al., 2019; Roberts et al., 2020). Experimental studies support overlap in PTSD-related pathophysiology and mechanisms contributing to cardiometabolic diseases (Miller et al., 2011). As such, PTSD is not only a brain disorder, but also a systemic illness with extensive downstream physical consequences (Mellon et al., 2018; Michopoulos et al., 2016), including metabolic alterations.

Metabolites are small, metabolizable molecules in circulation, such as lipids or amino acids (Wishart et al., 2022). As the endpoint along the ‘omics cascade’ most proximal to behaviors and diseases, they capture information beyond upstream genetic and transcriptomic regulations (Clish, 2015; Patti et al., 2012). Metabolic alterations may emerge following the onset of PTSD through multiple pathways, such as dysregulated hypothalamic–pituitary–adrenal axis functioning that may impair glucose signaling and metabolism (Cohen et al., 2009) and increased inflammatory responses and mitochondrial dysfunctions (Mellon et al., 2018). Studies on PTSD and metabolites have mostly focused on *a priori* selected candidates, such as steroids and steroid derivatives implicated in glucocorticoid metabolism, yielding null or inconsistent findings (Zhu et al., 2022). Another approach to metabolomic analyses is an agnostic investigation of all quantified metabolites in a biological system or specimen (Dunn et al., 2005). Four agnostic metabolomic studies of PTSD to date (Karabatsiakis et al., 2015; Konjevod et al., 2021; Kuan et al., 2022; Mellon et al., 2019) suggest that PTSD pathophysiology involves alterations of mitochondrial functioning, fatty acid metabolism, and lipid metabolism. However, compared with metabolomic studies of other psychological conditions (e.g., depression, Bot et al., 2020; Davyson et al., 2023), PTSD studies were relatively small (*N* = 38 to 204), with limited power to detect signals of individual metabolites, despite intriguing patterns in pathway- or module-level analyses. Furthermore, three studies included only male participants; no metabolomic analyses of PTSD have been performed in population-based cohorts of civilian women. Whether metabolic alterations are linked to remitted PTSD also remains poorly understood.

Metabolomic studies of depression and anxiety have established linkages between distress and metabolic alterations related to cardiometabolic health (Huang et al., 2020; Pu et al., 2020; van der Spek et al., 2023). Earlier work from our group identified and validated a metabolomic signature for chronic depression or anxiety in two cohorts of women (Shutta et al., 2021). We subsequently developed a metabolite-based distress score (MDS) composed of 20 plasma metabolites. This MDS was associated with increased risk of incident cardiovascular disease (CVD) and diabetes in independent samples (Balasubramanian et al., 2023; Huang et al., 2023). Given that PTSD is highly co-morbid and shares similar symptoms to both anxiety and depression and has also been reliably associated with adverse cardiometabolic health (Galatzer-Levy et al., 2013), metabolic variations captured by the MDS may be observed among individuals with PTSD.

The current study leveraged plasma metabolomics data from three subsamples nested within an ongoing cohort of predominantly civilian women in the Nurses’ Health Study II (NHSII) to address two primary research questions. First, we assessed whether PTSD shares common metabolic alterations with depression and anxiety by testing associations of PTSD with the previously derived MDS and its individual components (Balasubramanian et al., 2023). Of note, the case–control sample of chronic distress used to develop the MDS did not overlap with our analytic sample. We hypothesized that (1) persistent PTSD symptoms would be associated with the MDS and its individual metabolite components and (2) remitted PTSD would not be associated with metabolite alterations relative to not having PTSD. Second, to identify other markers related to PTSD, we conducted an agnostic metabolome-wide investigation of 339 metabolites and a differential network analysis characterizing systems-level differences. By incorporating detailed assessments of PTSD, metabolomic markers, and covariates, this study provides greater insight into whether and how metabolic dysregulation occurs with PTSD, suggesting potential pathways explaining associations between PTSD and chronic diseases of aging.

## Methods and materials

### Study population

NHSII is an ongoing, prospective cohort of 116,429 female registered nurses residing in 14 US states at enrollment in 1989. Participants were 25 to 42 years old at baseline and had completed biennial follow-up surveys for over three decades. Analytic samples for the present study are from three independent subsamples of participants nested within NHSII, all of whom completed a questionnaire in 2008 assessing lifetime exposure to traumatic events and PTSD. Consistent data collection, quality control, and preprocessing protocols for both questionnaire-based and metabolomic data were implemented for all participants. The first subsample draws on recent cohort-wide harmonization of metabolomics data across sub-studies of various disease endpoints (hereafter referred to as the *NHSII merged dataset*), from which we identified 2550 samples from 2435 women across two blood collections (2276 in 1996–1999, 44 in 2010–2012 and 115 with blood samples taken at both collections). The second subsample draws on 204 women in the Mind–Body Study (*MBS*), an NHSII sub-study investigating connections between psychosocial factors and biological processes, oversampling women who reported childhood abuse (Huang et al., 2019), with metabolomics data from blood collections between 2013 and 2014. The third subsample (hereafter referred to as the *Severe Distress Sample*) draws on new metabolomic data from blood samples collected between 2010 and 2012 among 196 participants, oversampling women with significant PTSD exposure. See Supplement and Supplemental Figure S1 for detailed descriptions of the subsample and a study timeline.

This study was approved by the institutional review board of the Brigham and Women’s Hospital and Harvard T.H. Chan School of Public Health; participants’ completion of questionnaires implied informed consent per study protocol.

### PTSD and trauma measures

PTSD and trauma were assessed in 2008 using the same measures for all study subsamples. Lifetime trauma exposure was assessed using the 16-item modified Brief Trauma Questionnaire (Schnurr et al., 2002), capturing experiences of 15 potentially traumatic events (e.g., life threats or serious injury) and ‘a seriously traumatic event not already covered’ based on DSM-IV diagnostic criteria (American Psychiatric Association, 1994). Participants reported their first trauma exposure, worst trauma exposure (if they experienced multiple exposures), and if exposed, their ages at the first and worst trauma events. We created an indicator variable for trauma exposure (exposed/unexposed). Participants were considered unexposed to trauma at a given blood collection if their first trauma occurred after blood collection or if they reported no trauma exposures as of 2008. Participants were prompted to answer questions about PTSD symptoms if they indicated any trauma exposure, following the diagnostic criteria.

Lifetime and past-month PTSD symptoms were measured with the seven-item Short Screening Scale for DSM-IV PTSD (Breslau et al., 1999), referencing the worst trauma identified by the respondent. While formal data regarding the duration of PTSD symptom experience were not obtained, symptoms reported in 2008 likely persisted since the onset of PTSD (i.e., the time of worst trauma) and reflect a different experience of PTSD from that associated with remitted symptoms. According to a previous meta-analysis, over 50% of PTSD cases were unremitted (Morina et al., 2014). Among participants who provided blood samples between 1996 and 1999, the average age at worst trauma was 32.2 years, with the trauma occurring more than 10 years prior to blood collection. As a result, two PTSD variables were derived: *persistent PTSD symptoms* – the count of PTSD symptoms experienced within the past 4 weeks at the time of reporting in 2008 (range = 0–7), which was modeled as a continuous variable and *remitted PTSD –* modeled as a binary indicator capturing whether the participant experienced lifetime PTSD (reporting ≥4 symptoms) but did not report experiencing any past-month symptoms (Ratanatharathorn et al., 2022; Sumner et al., 2015). The binary cut-point of ≥4 symptoms has been shown to capture PTSD cases with a sensitivity of 85% and a specificity of 93% (Breslau et al., 1999). A sensitivity analysis in the Supplement includes an alternative approach characterizing persistent PTSD as a binary variable (defined by ≥4 persistent symptoms).

### Blood collection and metabolomic profiling

All blood samples were collected and processed following standard procedures; samples were stored at −130 C^o^ or lower. Plasma metabolomic profiling was performed at the Broad Institute of the Massachusetts Institute of Technology and Harvard using a platform comprising complementary liquid chromatography-tandem mass spectrometry (LC–MS)-based methods. Three hundred and thirty nine metabolites were available in all three subsamples. Technical details about metabolite selection, profiling, and quality control are described elsewhere (Paynter et al., 2018) and in the Supplement.

To assess metabolic alterations linked to depression and anxiety, we calculated a metabolite-based distress score (MDS), following prior work (Balasubramanian et al., 2023). Of note, 19 out of the 20 metabolites identified in the original publication are available in our analytic subsamples and therefore included in the MDS. See Supplement for details of the MDS development and individual metabolite weights.

### Covariate measures

Three sets of covariates are considered incrementally in the analyses. Unless stated otherwise, all covariates were taken from self-report assessments closest in time to the relevant blood collection for each subsample and queried in the same way. (1) Minimal model covariates: demographic variables and matching factors from the sub-studies that followed a case–control design, namely, age, race/ethnicity, menopausal status, fasting status at blood collection, and sub-study indicator (only applicable in the NHSII merged dataset); (2) medical covariates: use of statins or other lipid-lowering drugs, hormone therapy, hypertension history, and type 2 diabetes history; (3) biobehavioral covariates: diet quality (measured by the Alternative Healthy Eating Index (Chiuve et al., 2012)), physical activity (measured by the total metabolic equivalent task (MET) hours per week (Ainsworth et al., 1993)), alcohol intake (g/day), smoking status (current/not current), caffeine intake (mg/day), and body mass index (BMI). See Supplement for additional information on covariates.

### Statistical analysis

Metabolite levels were log-transformed and normalized within each lab batch prior to data analysis. The degree of missingness was minimal for most metabolites: only 42 (12%) of metabolites had more than 1% of the values missing (Supplemental Figure S2). Based on low levels of missingness and the likelihood that the missingness primarily arose from the true value falling below the limit of detection (i.e., missing not at random, Do et al., 2018), we imputed missing values as ½ of the minimum observed value. This imputation strategy is also consistent with analytic protocols followed by prior cohort publications (Huang et al., 2020; Li et al., 2020). We additionally assessed results based on an alternative Random Forest-based imputation method (Supplement).

As missingness in covariates was minimal (up to 4%, for alcohol intake), we performed complete-case analysis. For all analyses, persistent PTSD symptoms, remitted PTSD status, and trauma exposure were included simultaneously in each model as primary predictors for a comprehensive characterization of PTSD (see Supplement for coefficient interpretations). Analyses were incrementally adjusted for the three sets of covariates described earlier. We accounted for multiple testing by applying false discovery rate (FDR) adjustment to the *p*-values and presenting results passing the significance threshold of adjusted *p* < 0.05. All analyses were performed using *SAS* version 9.4 and *R* version 4.1.0.

First, we assessed whether metabolites previously linked to depression and anxiety were associated with PTSD and trauma exposure. Specifically, we pursued two sets of analyses: (a) testing the association with the MDS (*N* = 2006 with complete data on all 19 metabolites) and (b) testing metabolite-level associations for each of the 19 available metabolites in the MDS. To allow for variations in estimates across subsamples, we first performed separate analyses within each subsample using either generalized estimating equations (in the NHSII merged dataset to account for within-individual dependence across the two blood collections by specifying an exchangeable covariance structure) or linear regression (in MBS and the Severe Distress Sample). The three subsample-specific estimates were then combined using a random effects meta-analysis to allow for variations in the exposure effects. Cochran’s *Q*-statistics were calculated to assess between-study heterogeneity.

Second, we performed a metabolome-wide agnostic analysis to identify metabolic markers (that may not have been in the MDS) associated with PTSD and trauma exposures. Extending the metabolite-level analysis described above to all 339 available metabolites, we also meta-analyzed estimates across subsamples for the association between each metabolite and PTSD/trauma exposure (N’s ranged from 438 to 2779 per metabolite; 83% of metabolites have *N* > 2000). Following the agnostic analysis, we identified metabolite classes enriched for associations with persistent PTSD symptoms from the minimally adjusted meta-analyses and compared with enrichment analysis of depression/anxiety conducted using summary statistics reported in prior research (Shutta et al., 2021).

Third, we characterized systems-level metabolic differences linked to PTSD by constructing a differential network between participants who reported any versus no persistent PTSD symptoms (exposed versus unexposed) using the differential network analysis in genomics (DINGO) algorithm (Ha et al., 2015). The categorization of no persistent PTSD symptoms included women who reported remitted lifetime PTSD but no persistent symptoms, experienced trauma but no PTSD symptoms, or did not experience trauma (see Supplement for further clarifications). We constructed our differential network using the 29 metabolites associated with persistent PTSD symptoms in the minimally adjusted analysis. By analyzing the conditional dependencies between metabolite pairs and testing for differences between participants with versus without persistent symptoms, DINGO provides a connected, network-focused view complementary to the agnostic analyses of individual metabolites. In a DINGO network, nodes represent individual metabolites and undirected, weighted edges reflect the *difference* in partial correlations of a metabolite pair between participants with persistent symptoms relative to controls. To assess the importance of each metabolite in the network structure, we calculated three different centrality measures: hub centrality (relative influence of a node) (Kleinberg, 1999), betweenness centrality (number of shortest paths through the network that include the node (Brandes, 2001; Freeman, 1978)); and closeness centrality (average distance from each node to all the other nodes in the network (Freeman, 1978)). A node with high values in any or all metrics indicates a metabolite whose patterns of correlations with other metabolites have changed in the presence of persistent symptoms; such a metabolite may be a key driver of potential metabolic dysregulations.

More details on our analytic approaches are provided in the Supplement.

## Results

### Sample characteristics

Participants’ demographic and health characteristics are shown in Table 1. Overall, 27.7% of our analytic sample reported persistent PTSD symptoms in 2008, 8% reported no persistent symptoms but met diagnostic criteria for probable lifetime PTSD (i.e., PTSD in remission), 39.7% were trauma exposed but did not have PTSD, and 24.7% were unexposed to trauma. Women reporting persistent symptoms were more likely to be post-menopausal, as samples from the MBS and Severe Distress Sample were obtained during later blood collections, when participants were older and mostly post-menopausal. Women who reported persistent symptoms or had remitted PTSD had slightly higher BMI and were more likely to have a history of hypertension.

**Table 1.** Participant characteristics by PTSD and trauma status (total sample *N* = 2950), assessed at or near time of blood collection

	Full sample	With persistent PTSD symptoms^a^	With remitted probable lifetime PTSD	Trauma exposed without PTSD	Trauma unexposed	Missing (%)
	*n* = 2950	*n* = 817	*n* = 235	*n* = 1170	*n* = 728	
*Demographic and technical factors*						
Age at blood collection, year, M (SD)	47.3 (7.0)	47.7 (7.1)	47.1 (7.0)	47.4 (7.2)	46.7 (6.7)	0
Race/ethnicity: non-White, *N* (%)	67 (2.3)	17 (2.1)	2 (0.9)	30 (2.6)	18 (2.5)	0
Menopausal status, *N* (%)						0
Pre-menopausal	1810 (61.4)	465 (56.9)	145 (61.7)	727 (62.1)	473 (65.0)	
Post-menopausal	802 (27.2)	249 (30.5)	60 (25.5)	306 (26.2)	187 (25.7)	
Missing/Unknown	338 (11.5)	103 (12.6)	30 (12.8)	137 (11.7)	68 (9.3)	
Fasting status: non-fasting, *N* (%)	722 (24.5)	194 (23.7)	56 (23.8)	290 (24.8)	182 (25.0)	0
*Medical factors*						
Statin or other lipid lowering drug use, *N* (%)	331 (11.2)	111 (13.6)	29 (12.3)	119 (10.2)	72 (9.9)	0
Hormone therapy current use, *N* (%)	419 (14.5)	132 (16.4)	40 (17.3)	148 (13.0)	99 (13.7)	1.9
Hypertension history, *N* (%)	428 (14.5)	138 (16.9)	41 (17.4)	151 (12.9)	98 (13.5)	0
Type 2 diabetes history, *N* (%)	55 (1.9)	21 (2.6)	6 (2.6)	15 (1.3)	13 (1.8)	0
*Biobehavioral factors*						
Diet quality score^b^, M(SD)	50.5 (12.6)	51.3 (12.9)	50.2 (12.6)	50.5 (13.0)	49.7 (11.6)	0.2
Recreational physical activity in MET-hrs/wk^c^, M (SD)	19.3 (21.8)	18.2 (20.7)	21.4 (30.3)	19.9 (21.7)	18.9 (19.6)	0.8
Alcohol intake, g/day, M (SD)	4.3 (7.7)	4.0 (7.4)	3.9 (6.1)	4.4 (8.3)	4.5 (7.5)	4
Current smoker, *N* (%)	201 (6.8)	66 (8.1)	21 (8.9)	73 (6.2)	41 (5.6)	0
Caffeine intake, mg/day, M (SD)	218.9 (207.1)	222.8 (215.7)	217.3 (209.3)	222.9 (212.2)	208.5 (187.3)	0.5
Body mass index, M (SD)	25.9 (5.7)	26.5 (6.1)	26.5 (5.9)	25.8 (5.6)	25.1 (5.0)	0

a Women with *any* persistent PTSD symptoms are grouped together for the purpose of showing the covariate distribution in this table, but the continuous symptom count was included in modeling. Note that 199 women in this group (24.4%) met diagnostic criteria for probable PTSD, that is, endorsing four or more symptoms.

b Measured by the Alternative Healthy Eating Index (AHEI).

c Total metabolic equivalent task (MET) hours per week.

### Does PTSD share common metabolic alterations with depression and anxiety?

A minimally adjusted meta-analysis of the association between the MDS and PTSD/trauma exposure demonstrated a significant positive relationship between persistent PTSD symptoms and the MDS; for each additional persistent symptom, MDS increased by 0.08 SD on average (95% CI: [0.04, 0.13]) (Table 2). However, MDS was not associated with remitted PTSD (*β* = 0.14, [−0.12, 0.39]) or trauma (*β* = −0.06, [−0.38, 0.27]). The association with persistent symptoms remained significant after adjusting for medical and biobehavioral factors in Models 2 and 3.

**Table 2.** Associations between PTSD exposures and the metabolite-based distress score

Exposure	Model 1: Minimal		Model 2: Medical		Model 3: Biobehavioral	
	*β* (95% CI)	*p-*value	*β* (95% CI)	*p-*value	*β* (95% CI)	*p-*value
*Random-effects meta-analysis* (*N* = 2950)						
Persistent PTSD symptom count	**0.08 (0.04, 0.13)**	**5.00E−04**	**0.08 (0.03, 0.12)**	**0.0011**	**0.07 (0.02, 0.11)**	**0.0036**
Remitted PTSD	0.14 (−0.12, 0.39)	0.2927	0.12 (−0.13, 0.37)	0.3367	0.1 (−0.14, 0.34)	0.4178
Trauma exposure	−0.06 (−0.38, 0.27)	0.7212	−0.03 (−0.32, 0.27)	0.8616	−0.1 (−0.42, 0.22)	0.5356
***NHSII merged dataset* (*N* = 2550)**						
Persistent PTSD symptom count	**0.06 (0.01, 0.12)**	**0.0252**	**0.06 (0.01, 0.12)**	**0.0313**	0.05 (−0.01, 0.1)	0.0831
Remitted PTSD	0.11 (−0.17, 0.39)	0.4494	0.12 (−0.16, 0.4)	0.4146	0.05 (−0.23, 0.32)	0.7466
Trauma exposure	0.13 (−0.02, 0.28)	0.0969	0.14 (−0.02, 0.29)	0.078	0.09 (−0.06, 0.23)	0.258
***Mind–Body Study* (*N* = 204)**						
Persistent PTSD symptom count	**0.18 (0.03, 0.33)**	**0.018**	**0.17 (0.03, 0.31)**	**0.0196**	**0.16 (0.02, 0.29)**	**0.0265**
Remitted PTSD	0.77 (−0.19, 1.72)	0.1164	0.64 (−0.27, 1.55)	0.1679	0.7 (−0.17, 1.57)	0.1144
Trauma exposure	−0.23 (−0.82, 0.36)	0.441	−0.24 (−0.8, 0.32)	0.405	−0.18 (−0.73, 0.36)	0.5122
***Severe distress sample* (*N* = 196)**						
Persistent PTSD symptom count	0.1 (−0.01, 0.2)	0.0662	0.08 (−0.02, 0.18)	0.1231	0.08 (−0.02, 0.17)	0.11
Remitted PTSD	−0.03 (−0.68, 0.62)	0.9345	−0.11 (−0.74, 0.52)	0.7321	0.07 (−0.53, 0.67)	0.8237
Trauma exposure	−0.33 (−0.84, 0.18)	0.2008	−0.25 (−0.74, 0.24)	0.3261	−0.41 (−0.88, 0.06)	0.0893

Persistent PTSD symptoms, lifetime PTSD in remission, and lifetime trauma exposure are included in the models simultaneously. Bold face indicates statistical significance (*p* < 0.05).

Model 1 covariates: age, race/ethnicity, menopausal status, fasting status at blood collection, and sub-study indicator (only applicable in the NHSII Merged Dataset); Model 2 covariates: Model 1 + use of statins or other lipid lowering drugs, hormone therapy, hypertension history, and type 2 diabetes history; Model 3 covariates: Model 2 + diet quality, physical activity, alcohol intake, smoking status, caffeine intake, and body mass index.

**Figure 1.**
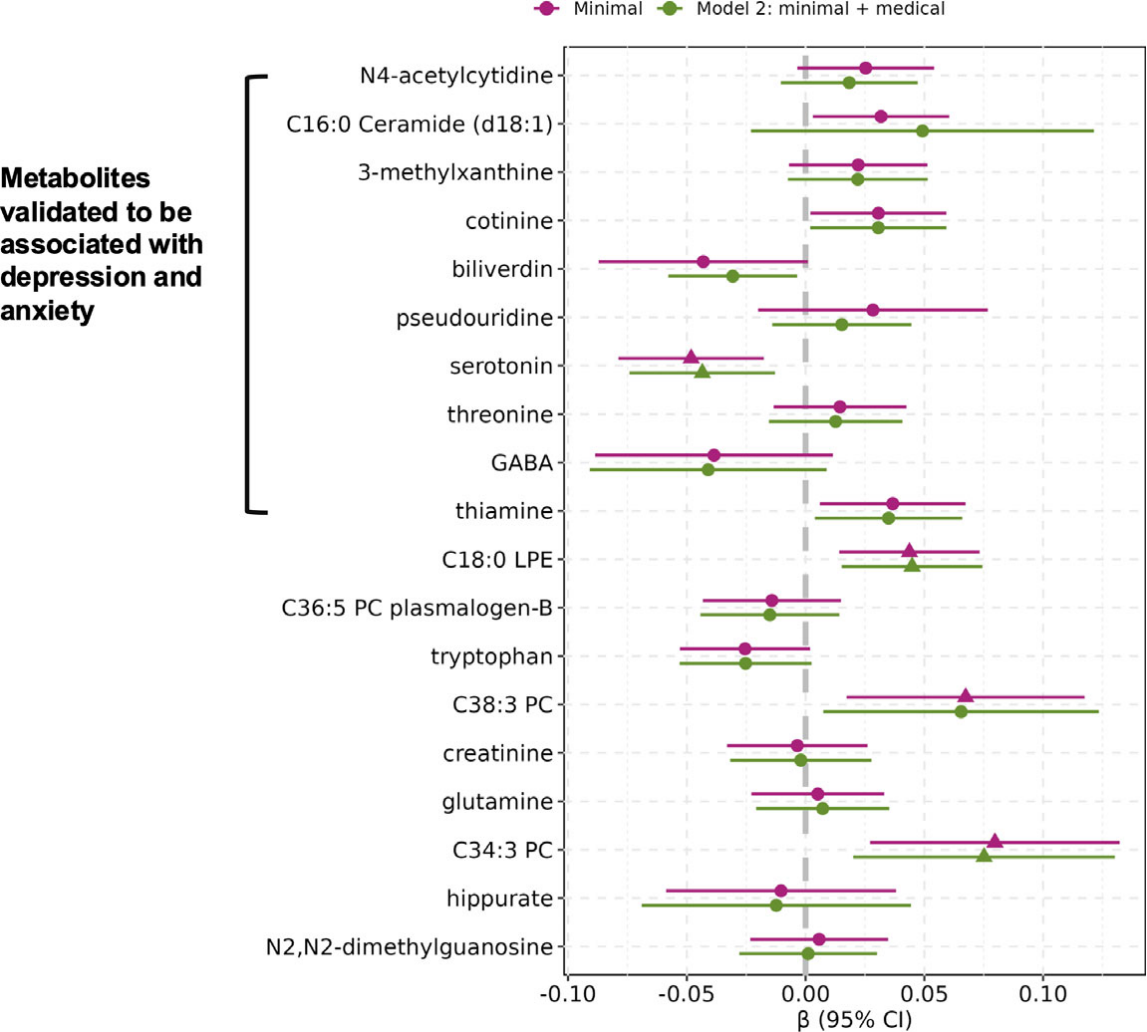
Associations between persistent PTSD symptoms and 19 metabolites previously associated with depression and anxiety included in the metabolite-based distress score (MDS), adjusting for baseline covariates (minimal, or Model 1) and medical factors (Model 2). Estimates are obtained from random effects meta-analyses of all three subsamples. For the β estimates, triangles indicate statistically significant associations after false discovery rate corrections for the number of metabolites tested (19; FDR adjusted *p* < 0.05) and circles indicate associations not statistically significant. Statistical model: metabolite-based distress score ~ Persistent PTSD symptom count + remitted PTSD + trauma exposure + covariates. Model 1 (minimal) covariates: age, race/ethnicity, menopausal status, and fasting status at blood collection. Model 2 covariates: Model 1 + use of statins or other lipid lowering drugs, hormone therapy, hypertension history, and type 2 diabetes history.

Four individual metabolites comprising the MDS were significantly associated (FDR < 0.05, correcting for 19 tests) with higher persistent PTSD symptoms in minimally adjusted models, and three remained significant after accounting for medical factors (Model 2 in Figure 1 and Supplemental Table S1): lower serotonin (*β* = −0.04 [−0.07, −0.01]) and higher levels of two glycerophospholipid metabolites (C18:0 LPE: *β* = 0.04 [0.02, 0.07]; C34:3 PC: *β* = 0.08 [0.02, 0.13]). Directions of these associations were consistent with previously reported associations with depression or anxiety. No associations were found with remitted PTSD or trauma exposure (Supplemental Tables S1B and C).

In the metabolite set enrichment analysis of metabolite classes based on metabolome-wide results, glycolipids and glycerophospholipids were significantly enriched for positive associations with persistent PTSD symptoms (Supplemental Figure S3). However, glycerophospholipids were enriched for inverse associations with depression/anxiety. Sphingolipids, imidazopyrimidines, and steroids were significantly enriched for associations with depression/anxiety; while the directions of enrichment for these three classes were the same with persistent PTSD symptoms, they did not reach significance after FDR corrections.

### Are unique metabolic markers related to PTSD symptoms?

**Figure 2.**
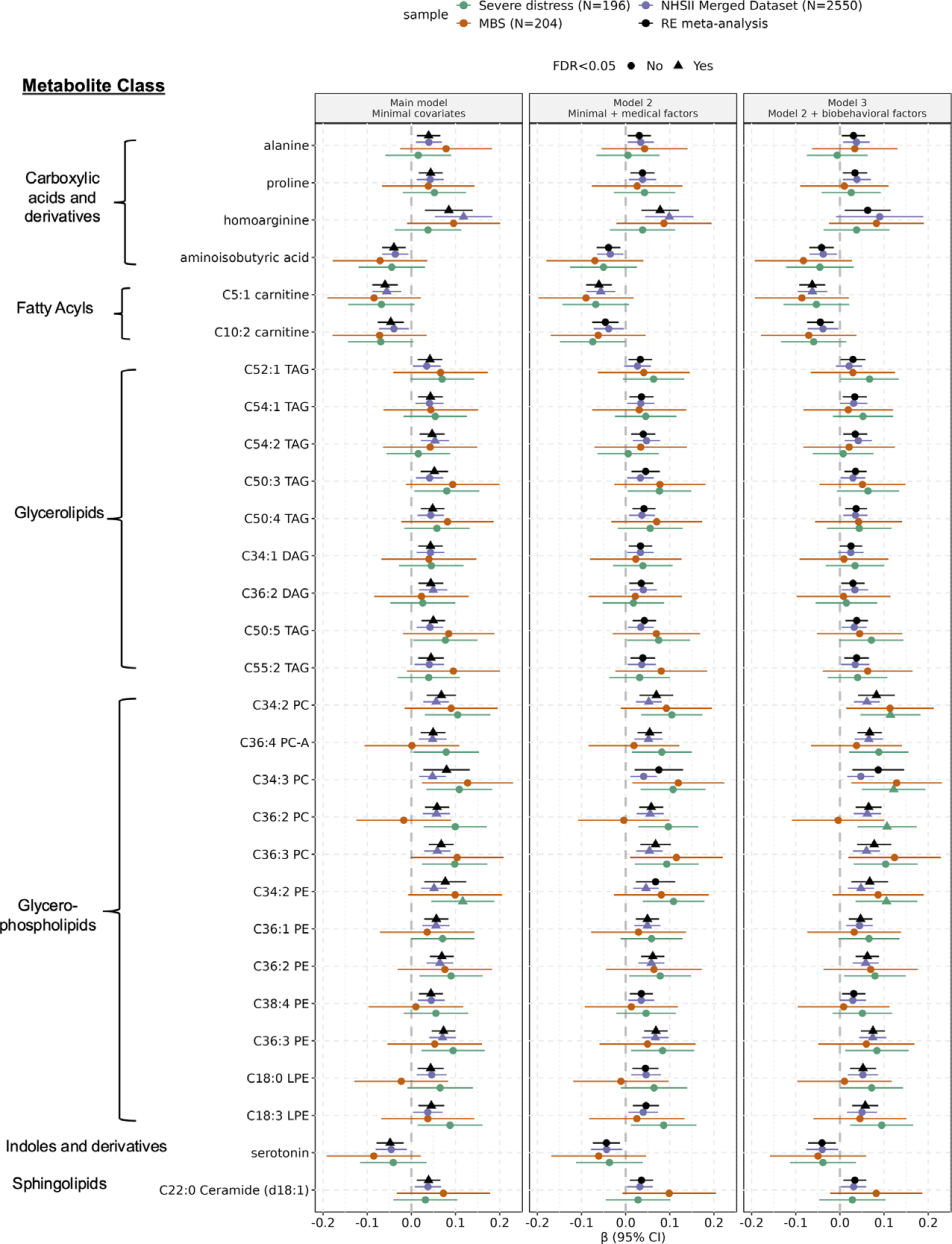
Agnostic per metabolite analysis results: statistically significant associations between persistent PTSD symptoms and 29 metabolites in the random-effects meta-analysis, after adjusting for technical covariates (Model 1) and multiple testing corrections. Estimates are obtained from both random effects meta-analyses of all three subsamples (black), and individual sub-samples: NHSII merged dataset (purple), the Mind–Body Study (MBS; orange), and the Severe Distress Sample (green). For the β estimates, triangles indicate statistically significant associations after false discovery rate corrections for the number of metabolites tested (339; FDR adjusted *p* < 0.05) and circles indicate associations not statistically significant. Statistical model: metabolite ~ Persistent PTSD symptom count + remitted PTSD + trauma exposure + covariates. Model 1 (minimal) covariates: age, race/ethnicity, menopausal status, fasting status at blood collection, and sub-study indicator (only applicable in the NHSII Merged Dataset); Model 2 covariates: Model 1 + use of statins or other lipid lowering drugs, hormone therapy, hypertension history, and type 2 diabetes history; Model 3 covariates: Model 2 + diet quality, physical activity, alcohol intake, smoking status, caffeine intake, and body-mass index.

In agnostic analyses evaluating 339 metabolites (including metabolites in the MDS), 29 were significantly linked to persistent PTSD symptoms in the minimally adjusted (Model 1) meta-analyses after multiple testing corrections (FDR < 0.05; Model 1 in Figure 2), and no metabolites were linked to trauma exposure or remitted PTSD (Supplemental Table S2). Nine metabolites remained significantly associated with persistent PTSD symptoms after accounting for medical factors, and 11 remained significant after additionally adjusting for biobehavioral factors (Figure 2 and Supplemental Table S2).

None of the 29 metabolites showed significant between-sample heterogeneity (*p* > 0.1 for Cochran’s *Q*), despite age-associated differences in demographic and health characteristics between subsamples. Twenty-five metabolites were positively associated with persistent symptoms, and four were inversely associated. Only three out of the 29 metabolites were among the previously identified metabolites for the MDS. The magnitude of coefficients for each additional persistent symptom ranged from 0.04 (95% CI [0.01, 0.07], C22:0 Ceramide) to 0.08 (95% CI [0.03, 0.14], homoarginine). The most common metabolite classes represented among these 29 metabolites were glycerophospholipids (12 metabolites) and glycerolipids (nine metabolites).

### Differential network analysis

**Figure 3.**
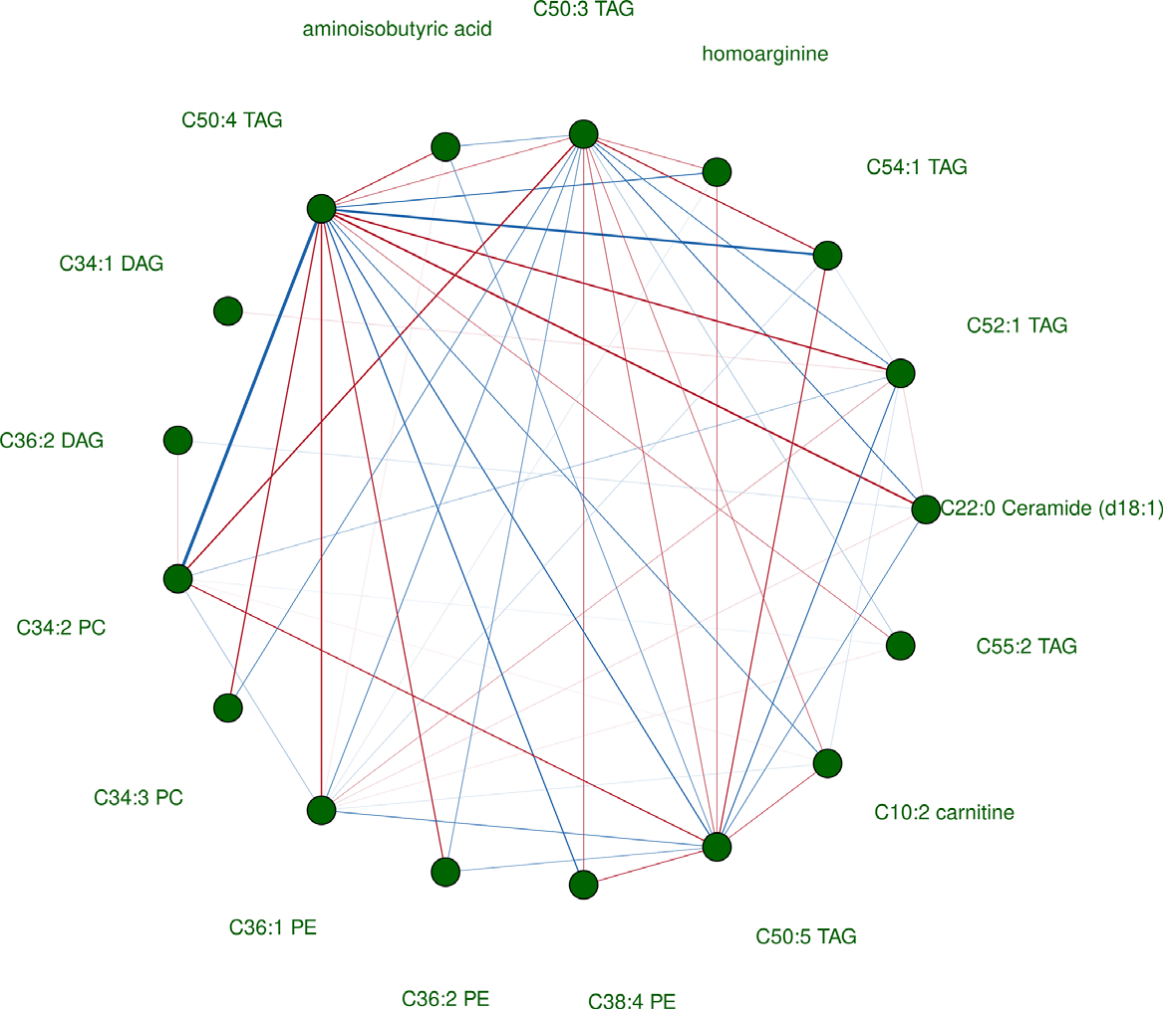
Differential network showing statistically significant differences in partial correlations estimated between 29 metabolites associated with persistent PTSD symptoms in the meta-analysis of the minimal model. Seventeen metabolites connected to 54 significant edges after multiple testing corrections (FDR < 0.05) are shown in the figure. Analysis was performed within a subsample of 858 independent participants who had complete data on all 29 metabolites selected based on the minimally adjusted agnostic analysis. The differential network was constructed by comparing participants reporting persistent PTSD symptoms in 2008 (*N* = 272) and participants who did not report persistent symptoms regardless of trauma exposure status (*N* = 586). Red edges indicate that partial correlations between pairs of metabolites were higher among participants with persistent symptoms versus without. Blue edges indicate that partial correlations between pairs of metabolites were lower among participants with persistent symptoms versus without. The width of edges corresponds to the weight, that is, difference in partial correlations between individuals with persistent PTSD symptoms versus without.

Informed by the agnostic analysis where associations were evident only with respect to persistent PTSD symptoms in all models, we estimated a differential network comparing participants with (*N* = 272) versus without (*N* = 586) any persistent PTSD symptoms (Figure 3). Out of 29 metabolites included in the analysis, 17 with 54 significant edges (FDR < 0.05) between them were retained in the graph. Half of the significant edges were positive, suggesting that the partial correlations between metabolite pairs were higher among participants with versus without persistent symptoms, with the largest difference observed between C50:4 TAG and C34:2 PC (Δ_partial r_ = 0.16). Among the negative edges (i.e., edges representing lower partial correlations among participants with persistent symptoms), the largest difference was observed between C22:0 Ceramide (d18:1) and C50:4 TAG (Δ_partial r_ = −0.09). Summary results of network measures highlight the importance of a triglyceride, C50:4 TAG, in the network of metabolite differential partial correlations that potentially emerged as a result of having persistent PTSD symptoms, which had the highest hub score, betweenness, and closeness (Supplemental Table S3).

## Discussion

Our study is the first large-scale metabolomics analysis of PTSD in a predominantly civilian sample of middle-aged women. We found overlap as well as notable differences in metabolic alterations linked to PTSD in comparison to metabolites previously linked with depression or anxiety in this population. We identified 29 metabolites associated with persistent PTSD symptoms and most were not linked to depression and anxiety in prior research. Patterns of correlations in a metabolite network suggest that triglycerides may be key markers in metabolomic differences associated with persistent PTSD.

Consistent with prior metabolomic investigations of distress, the current study highlights role of phospholipid dysregulation in persistent PTSD. Phospholipids form the backbone of neural membranes, maintaining membrane functions and transmembrane signaling in the brain (Farooqui et al., 2004). A recent mouse study found that mice characterized as being susceptible to PTSD showed sustained increased hippocampal levels of glycerophosphoethanolamines (PEs) and phosphatidylcholine (PCs) (Pascual Cuadrado et al., 2022). Prior studies have also linked phospholipid dysregulation reflected in human peripheral tissues (e.g., blood) to higher risks of neurodegenerative diseases (González-Domínguez et al., 2014; Li et al., 2019) and inflammation (Leitinger, 2003). However, directions of associations between PTSD and phospholipid concentrations varied: similar to our findings, several studies identified elevated levels of phospholipids in smaller clinical and veteran samples (Karabatsiakis et al., 2015; Konjevod et al., 2021). However, two studies of male miliary samples revealed *decreased* levels of PEs and PCs in PTSD cases compared to healthy controls (Emmerich et al., 2016; Konjevod et al., 2022). Intriguingly, a recent lipidomic analysis in 80 men and women reported PEs as the only lipid subclass altered among women with PTSD, whereas 9 out of 13 subclasses were altered among men with PTSD (Bhargava et al., 2024). These differences may be due to variations in trauma type, study population, and analytic strategies; with data from a large, civilian sample, our study may capture subclinical variations in PTSD symptom severity and nuances in associated metabolomics profiles. Longitudinal investigations in well-characterized, large samples including men and women may better characterize lipid dysregulations following the development of PTSD, examine potential causal relationships, and identify key effect modifiers, such as gender differences in trauma responses (Olff, 2017). Taken together, PTSD may give rise to phospholipid dysregulations with profound impacts on cellular functioning and inflammatory responses, though the specific molecules and directions of associations need further validation. The prominent role of C50:4 TAG as highlighted in our differential network analysis indicates that triglycerides may act as key regulators of changes in lipid pathways triggered by persistent PTSD symptoms, including disruptions in phospholipid metabolism.

Another novel contribution of our study is identifying a positive association with homoarginine and an inverse association with C5:1 carnitine in relation to persistent PTSD symptoms. The positive association with homoarginine may reflect an increase in nitric oxide formation and inflammation occurring with PTSD (Oosthuizen et al., 2005), as homoarginine promotes synthesis of nitric oxide and increases oxidative stress (Bernstein et al., 2015; Lambert et al., 1992). Some carnitines, namely, pentadecanoylcarnitine, have also shown endocannabinoid-like activities and been implicated in regulating inflammation and mood (Venn-Watson et al., 2022). In a pilot study of chronically stressed female mice, carnitines were over-expressed in plasma but under-expressed in ovarian tissues, highlighting the dynamic processes of uptake or release of carnitine metabolites under stress in different tissues (Zeleznik et al., 2022). Our findings suggest that homoarginine and C5:1 carnitine may be promising targets for follow-up experiments of stress response pathophysiology.

The substantial evidence for metabolite associations with persistent PTSD symptoms but not trauma exposure or remitted PTSD suggests chronic, non-remitted symptoms may have more profound metabolic consequences. Similarly, PTSD research examining other biomarkers shows elevated inflammation in current PTSD cases but fewer differences between remitted PTSD cases and controls without PTSD (Glaus et al., 2018; O’Donovan et al., 2017). However, the lack of associations with trauma or remitted PTSD in our study should be interpreted with the comparison groups in mind: our modeling strategies led to a comparison of women who were trauma-exposed but reported no lifetime PTSD to women who reported no trauma exposure. Prior work examining trauma exposure more broadly (regardless of PTSD symptoms) has identified metabolite associations with trauma and suggested the mediating roles of psychological distress (Huang et al., 2022). Furthermore, the sample size of women with remitted PTSD was relatively small (*N* = 235); as such, our analyses may be underpowered to detect metabolomic associations with remitted PTSD.

Our comparison of PTSD-related metabolic alterations with other highly comorbid chronic distress phenotypes, namely, depression or anxiety, suggests that some alterations in metabolic markers may be specific to each disorder. In clinical presentations, while all chronic distress may be characterized by high levels of negative affect or dysphoria, trauma-related avoidance and hypervigilance are distinctive features that mark PTSD (Post et al., 2011). Moreover, substantial overlap exists between known underlying biological changes occurring with PTSD and depression, including structural and functional changes in the brain and endocrine responses (Ploski & Vaidya, 2021). Yet notable distinctions exist as well, such as enhanced glucocorticoid sensitivity in PTSD versus greater glucocorticoid resistance in major depression (Yehuda, 2001). Our findings shed light on shared metabolic features across disorders, such as decreased serotonin and relevance of phospholipid metabolism (Lin et al., 2010). We also highlight the possibility of disorder-specific markers, such as elevated levels of glycerolipid and glycerophospholipid metabolites in PTSD, and dysregulations of amino acids and fatty acids occurring with depression and anxiety (Shutta et al., 2021). These disorder-specific metabolic differences may capture downstream molecular changes linked to distinctive symptom clusters such as avoidance; although we did not have available data to examine symptom-specific patterns, more granular investigations could further our understanding of connections between chronic distress and metabolic dysregulations beyond diagnoses. Understanding whether these distinctions between disorders replicate across populations and identifying transdiagnostic metabolomic profiles of distress are also critical next steps (Pinto et al., 2017; Waszczuk et al., 2023), especially considering the challenges in validating biomarkers for major psychiatric disorders (Abi-Dargham et al., 2023).

These findings may provide further insight into mechanisms underlying elevated cardiometabolic disease and mortality risks associated with PTSD (Levine et al., 2013; Roberts et al., 2020). In a recent analysis of plasma metabolites associated with mortality among over 11,000 US adults, 11 out of the 29 metabolites linked to PTSD symptoms in our study also showed positive associations with higher all-cause mortality risk (Wang et al., 2023). Immune alterations in response to traumatic stress may contribute to lipid accumulation (Mellon et al., 2018); phospholipid metabolism dysregulations could cause mitochondrial dysfunction and impaired insulin signaling, perpetuating a vicious cycle leading to elevated risk of cardiometabolic diseases (Meikle & Summers, 2017). While diet and pharmacological agents have been the most studied intervention targets, the prominent lipid associations with PTSD suggest that preventing, screening, and treating psychological distress could be another strategy for improving cardiometabolic health (Levine et al., 2021).

Our study has several notable strengths. First, most existing metabolomic studies of PTSD were in male, military, and/or highly selected clinical samples (Zhu et al., 2022); our findings address an important gap by examining a large, population-based sample of predominantly civilian women. Second, we strengthened the methodological rigor of our study by leveraging detailed covariate data collected over decades and combining measures from blood samples collected at different time points relative to PTSD assessments. Nonetheless, several limitations should be considered. First, the NHSII cohort consists of predominantly White, professional women; thus, our findings may not generalize to populations experiencing more severe forms of trauma or PTSD symptoms and women with non-White racial and ethnic backgrounds. Relatedly, although age-dependent between-sample heterogeneity was not observed according to the Cochran’s *Q*-tests, these tests may be underpowered with a small number of sub-studies (Gavaghan et al., 2000). Therefore, we cannot rule out potential effect modification by age or other demographic characteristics. Third, our measures of trauma and PTSD may not fully capture the complexity of these experiences. Instead of structured clinical interviews, PTSD symptoms were evaluated using self-reported data from the seven-item Short Screening Scale (Breslau et al., 1999). Furthermore, trauma and PTSD measures were queried only in 2008 whereas most blood samples were collected between 1996 and 1999; while most participants reported exposure to their worst trauma (used to date PTSD onset) occurred at least 10 years prior to the date of the blood collection, errors in recall could still influence our results. For women whose blood samples were collected after 2008, exposure misclassification was also a concern because no PTSD information was available after 2008. To mitigate these potential biases, we included samples with later blood collections when possible, carefully dated PTSD onset by checking the reported age of trauma exposure, and interpreted our findings cautiously. Nonetheless, the identified associations may reflect reverse causation whereby changes in metabolite levels increased PTSD susceptibility. Moreover, we cannot fully disentangle effects of PTSD and depression on metabolomic profiles: depression and PTSD are highly comorbid and identifying their separate effects would require being able to specify a temporal causal relationship between the onset of these conditions and test this causal model using repeated assessments of PTSD and depression, which were unavailable in our study. Given the constraints, we focused on comparing the metabolites associated with PTSD versus metabolites linked to depression in previous publications using independent samples.

Through large-scale plasma metabolomic profiling of PTSD among middle-aged women, we identified several promising metabolic markers associated with having persistent PTSD symptoms. Metabolic dysregulations, in particular elevated lipid levels, may occur with PTSD and be partly responsible for associations of PTSD with adverse physical health outcomes. With further research, screening for and treating chronic distress disorders like PTSD could be a crucial intervention for improving health among trauma-exposed women.

## Supporting information

Zhu et al. supplementary material 1 Zhu et al. supplementary material

Zhu et al. supplementary material 2 Zhu et al. supplementary material

## Supplementary material

The supplementary material for this article can be found at http://doi.org/10.1017/S0033291724003374.
