# Persistent PTSD symptoms are associated with plasma metabolic alterations relevant to long-term health: A metabolome-wide investigation in women - Erratum

**DOI:** 10.1017/S0033291725000339

**Published:** 2025-02-20

**Authors:** Yiwen Zhu, Katherine H. Shutta, Tianyi Huang, Raji Balasubramanian, Oana A. Zeleznik, Clary B. Clish, Julián Ávila-Pacheco, Susan E. Hankinson, Laura D. Kubzansky

**Affiliations:** 1 Department of Epidemiology, Harvard T.H. Chan School of Public Health, Boston, MA, USA; 2 Department of Biostatistics, Harvard T.H. Chan School of Public Health, Boston, MA, USA; 3 Channing Division of Network Medicine, Department of Medicine, Brigham and Women’s Hospital and Harvard Medical School, Boston, MA, USA; 4 Harvard Medical School, Boston, MA, USA; 5 Department of Biostatistics and Epidemiology, School of Public Health and Health Sciences, University of Massachusetts Amherst, Amherst, MA, USA; 6 Broad Institute of Massachusetts Institute of Technology and Harvard, Cambridge, MA, USA; 7 Department of Social and Behavioral Sciences, Harvard T.H. Chan School of Public Health, Boston, MA, USA

When this article was originally published in Psychological Medicine it omitted to include Dr. Susan E Hankinson as a co-corresponding author alongside Yiwen Zhu. This has now been updated.

The publisher apologises for this error.
